# Small but Powerful: The Human Vault RNAs as Multifaceted Modulators of Pro-Survival Characteristics and Tumorigenesis

**DOI:** 10.3390/cancers14112787

**Published:** 2022-06-03

**Authors:** Stefano Gallo, EunBin Kong, Iolanda Ferro, Norbert Polacek

**Affiliations:** 1 Department of Chemistry, Biochemistry and Pharmaceutical Sciences, University of Bern, 3012 Bern, Switzerland; stefano.gallo@unibe.ch (S.G.); eunbin.kong@unibe.ch (E.K.); iolanda.ferro@roche.com (I.F.); 2 Graduate School for Cellular and Biomedical Sciences, University of Bern, 3012 Bern, Switzerland

**Keywords:** non-coding RNA, vault RNA, vault particle, major vault protein, tumorigenesis, apoptosis resistance, drug resistance

## Abstract

**Simple Summary:**

Small non-protein-coding RNAs have been recognized as valuable regulators of gene expression in all three domains of life. Particularly in multicellular organisms, ncRNAs-mediated gene expression control has evolved as a central principle of cellular homeostasis. Thus, it is not surprising that non-coding RNA misregulation has been linked to various diseases. Here, we review the contributions of the four human vault RNAs to cellular proliferation, apoptosis and cancer biology.

**Abstract:**

The importance of non-coding RNAs for regulating gene expression has been uncovered in model systems spanning all three domains of life. More recently, their involvement in modulating signal transduction, cell proliferation, tumorigenesis and cancer progression has also made them promising tools and targets for oncotherapy. Recent studies revealed a class of highly conserved small ncRNAs, namely vault RNAs, as regulators of several cellular homeostasis mechanisms. The human genome encodes four vault RNA paralogs that share significant sequence and structural similarities, yet they seem to possess distinct roles in mammalian cells. The alteration of vault RNA expression levels has frequently been observed in cancer tissues, thus hinting at a putative role in orchestrating pro-survival characteristics. Over the last decade, significant advances have been achieved in clarifying the relationship between vault RNA and cellular mechanisms involved in cancer development. It became increasingly clear that vault RNAs are involved in controlling apoptosis, lysosome biogenesis and function, as well as autophagy in several malignant cell lines, most likely by modulating signaling pathways (e.g., the pro-survival MAPK cascade). In this review, we discuss the identified and known functions of the human vault RNAs in the context of cell proliferation, tumorigenesis and chemotherapy resistance.

## 1. Introduction

The central dogma of molecular biology that states that the flow of genetic information is always conveyed from the DNA to the RNA level and subsequently transferred to proteins was proposed more than 60 years ago [1]. To date, this dogma is still widely accepted, but currently, the picture appears more complex and diversified. At the turn of the century, with the finalization of the human genome reference sequence, it immediately became clear that many of the information encoded in the genome of an organism are not directly related to protein synthesis [2,3]. In multicellular eukaryotes such as humans, the vast majority of the genome is transcribed into RNA, and increasing evidence suggest that some of these transcripts have evolved for regulatory purposes [4,5]. Many genomic loci, despite being transcribed into RNA molecules, lack translation potential, yet they contribute to cell homeostasis. These transcripts are named non-coding RNAs (ncRNAs). In recent years, also due to the advent of high throughput sequencing technologies, many regulatory ncRNA have been detected and characterized [6]. Within the heterogeneous group of ncRNA, it is possible to distinguish several classes including ribosomal RNAs (rRNAs), transfer RNAs (tRNAs), tRNA-derived small RNAs (tDRs or tRFs), microRNAs (miRNAs), small interfering RNAs (siRNAs), prokaryal short antisense RNAs (sRNAs), CRISPR RNAs (crRNAs), small nuclear RNAs (snRNAs), small nucleolar RNAs (snoRNAs), circular RNAs (circRNAs), long ncRNAs (lncRNAs), long intergenic ncRNAs (lincRNAs), piwi-interacting RNAs (piRNAs), small Cajal body RNAs (scaRNAs), vault particle-associated RNAs (vtRNAs) or extracellular RNAs (exRNAs). Some ncRNAs, such as rRNAs and tRNAs, are essential for protein biosynthesis and are well characterized in all domains of life [7,8,9]. Furthermore, in eukarya, we have a good understanding of the molecular mechanisms and function of miRNAs and some lncRNAs [10,11,12]. Generally, ncRNAs control all sorts of cellular mechanisms but most importantly gene expression at the transcriptional and post-transcriptional levels. Indeed, ncRNAs have major roles in chromatin remodeling, DNA methylation and gene silencing, in addition to splicing, and mRNA stability [13,14,15,16,17]. Additionally, ncRNAs can interfere with signaling pathways and, therefore, orchestrate inter- and intra-cellular communication [18,19]. Since ncRNAs play a crucial role in the regulation of several cellular mechanisms, they are consequently often involved in disease and cancer onset [20,21,22,23,24,25].

**Figure 1:**
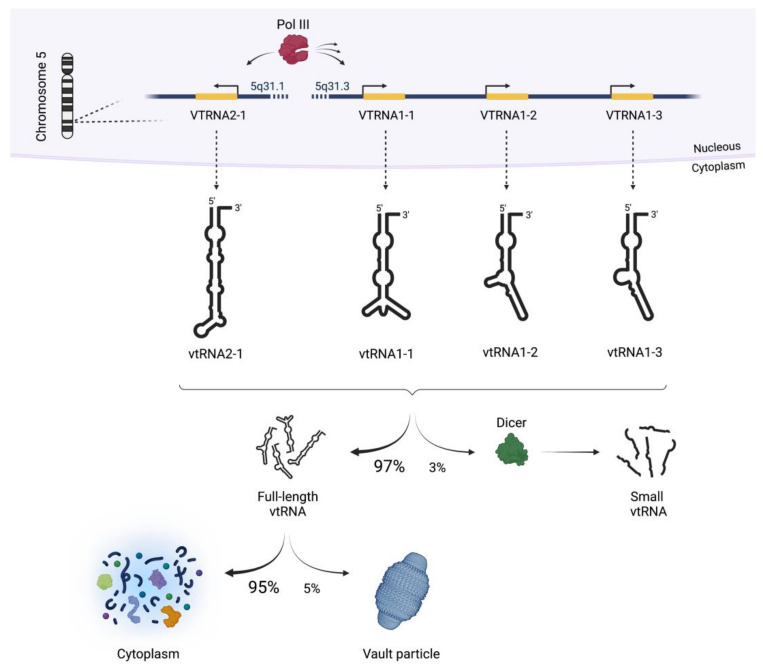
The human vault RNAs. The four human vault RNAs (vtRNAs) are encoded on two loci on chromosome 5 and are expressed by RNA polymerase III (Pol III). A minor fraction of the vtRNAs can be further processed by Dicer into small vtRNA fragments (~2–5% as shown for vtRNA2-1 [45]). The vast majority of full length vtRNAs locates to and functions in the cytoplasm (~95%), whereas about 5% associate with the 13 MDa large vault particle [32,46].

Similarly to other ncRNAs, vtRNAs have been reported to regulate several cellular pathways and are, thus, involved in cell homeostasis and tumorigenesis [26]. vtRNAs are molecules of about 100 nucleotides (nt) initially described four decades ago as part of a very large, hollow and barrel-shaped ribonucleoprotein (RNP) named the vault complex. The vault RNP is a 13 MDa mainly cytoplasmic complex and is predominantly composed of proteins. In mammals, the vault complex consists of three proteins: the 104 kDa major vault protein (MVP), the 193 kDa vault poly (ADP-ribose) polymerase (VPARP) and the 240 kDa telomerase-associated protein-1 (TEP1) [27,28,29,30,31]. As the name suggests, MVP is the dominating component and represents over 70% of the vault particle mass, while the associated vtRNAs contribute less than 5% [27]. vtRNAs are not only components of the vault complex and in fact the vast majority of them (95%) are not associated with vaults and are distributed in the cytoplasm while engaging in other cellular processes [32,33]. The four human vtRNAs are encoded on chromosome 5q31 (Figure 1), whereas three are located in the *VTRNA-1* locus (vtRNA1-1, vtRNA1-2 and vtRNA1-3) and the fourth (vtRNA2-1) is encoded in a separate locus [34,35]. The *VTRNA* genes are transcribed by the RNA polymerase III (pol III); hence, they are under the control of pol III type 2 promoter. *VTRNA* genes feature two internal promoter sequences, namely A box and B box, which enable the binding with the transcription factors TFIIIC and TFIIIB. These transcription factors provide pol III stability at the transcription start site and promote RNA synthesis [34,36]. However, the promoters of the *VTRNA-1* and *VTRNA-2* loci are not identical. Consequently, the expression efficiencies of different *VTRNA* genes are not always comparable [34,37,38,39,40,41].

Evolutionarily, the vault complex is a highly conserved RNP found in many eukaryotic species, from deuterostomes to slime mold. However, it is missing in many common model organisms including *C. elegans*, *D. thaliana*, *D. melanogaster* and *S. cerevisiae* [34,42,43]. Moreover, despite vtRNAs sequences being highly conserved, the number of paralogues vary across the animal kingdom. In addition to the four vtRNA paralogues, the human genome contains one confirmed pseudogene on the X chromosome named *VTRNA3-1P* [34] and another unconfirmed pseudogene on chromosome 2 named *VTRNA2-2P*. The human *VTRNA1* locus encodes three paralogues, and their orthologues can also be found in the phylogenetically close chimp (*P. troglodytes*) genome. Instead, only two *VTRNA1* orthologues are encoded in the genome of other primates. This evidence, together with sequence alignment analysis, revealed that *VTRNA1-2* and *VTRNA1-3* are the consequence of a very recent gene duplication event. Indeed, most mammals, including the relevant model organism mouse (*M. musculus*), have only a single *VTRNA1* copy. In most mammals, the *VTRNA2* locus either is missing or encodes for only one vtRNA paralogue, with the exception of the sloth (*C. hoffmanni*) genome in which two *VTRNA2* paralogues are found [34].

Generally, vtRNAs are around 100 nt structured RNAs, but their length is species-specific and can be as short as 88 nt, similarly to some human vtRNAs, and as long as 143 nt in the case of the single mouse vtRNA. Furthermore, vtRNA secondary structure is well conserved and consists of an extended stem structure in which the 3′ end connects to the 5′ end of the molecule and a central domain of variable structure (Figure 1). Such structure is also referred to as the “panhandle”; in different animals, the handle portion is conserved and its length is consistent, whereas the sequence and size of the central domain are highly variable [34]. Moreover, there is evidence of the further processing of vtRNA into smaller molecules of about 23 nt in length [44,45]. To date, few examples of vtRNA-derived small RNAs have been studied experimentally; however, it is possible that they operate in an miRNA-like fashion.

Here, we review investigations on human vtRNAs, outlining their possible functions in cell metabolism regulation, with an emphasis on their role in tumorigenesis, cell death and survival as well as in chemotherapy resistance. Among all human vtRNAs, vtRNA1-1 has been the most studied to date; hence, we more extensively review the functions of this paralogue. A more limited discussion is dedicated to the remaining three paralogues because of the current research gap in the vtRNA field. Moreover, since many studies did not separate the contribution of free vtRNA from vtRNA bound to the vault RNP, we additionally summarized the contribution of the vault complex and the MVP to cancer, including drug resistance, proliferation and apoptosis.

## 2. vtRNA1-1: Contributions to Cell Proliferation and Tumorigenesis

### 2.1. vtRNA1-1 and Cell Proliferation

Humans and some primates are the only animal species in which four vtRNAs are expressed. On the contrary, only one vtRNA is encoded in the genome of rodents and many other animals. Among the four vtRNAs expressed by humans, vtRNA1-1 is the most studied so far. This 98 nt long RNA constitutes 80% of the ribonucleic mass found in the vault RNP [47]. Despite this, only 5% of the expressed vtRNA1-1 actually associates with the vault particle, and consequently, 95% of the transcribed vtRNA1-1 is found to be non-associated with the 13 MDa vault complex in the cytoplasm (Figure 1) [32,46]. Because of its abundance in the “free” form, vtRNA1-1 was suspected to have important roles unrelated to the vault particle. 

**Figure 2:**
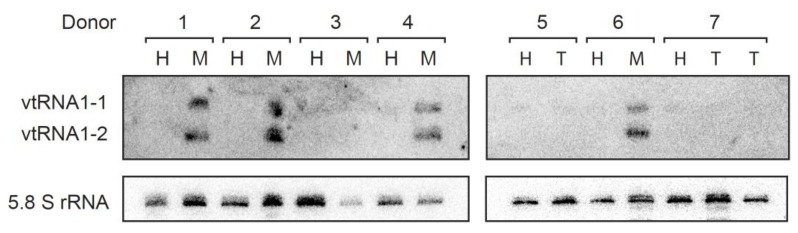
Vault RNAs are abundantly expressed in liver samples. Northern blot analysis in paired patient-derived tissues was performed as described in [53] and revealed that vtRNA1-1 and vtRNA1-2 are expressed in metastatic but not in non-pathological adjacent liver tissues; healthy (H), tumor (T) and metastasis (M). 5.8S rRNA serves as internal loading control.

In the last two decades vtRNA1-1 role was extensively investigated in human cell lines. In HeLa cells, which derive from cervical adenocarcinoma, the knock out (KO) of the vtRNA1-1 lead to a significant decrease in cell proliferation [48]. The proliferation defect following a vtRNA1-1 KO was also observed in the hepatocellular carcinoma cell line Huh-7 [49]. In both KO cell lines, vtRNA1-1 was re-introduced in the genome by lentiviral transduction, and these vtRNA1-1 complemented cell lines rescued the growth defect [48,49]. These studies revealed that, in cancer cells, vtRNA1-1 is an important proliferation factor and its absence impairs cell growth. In vivo support for this conclusion was obtained in patient-derived liver samples. Northern blot analysis revealed that vtRNA1-1, along with vtRNA1-2, was abundantly expressed in metastatic but not in non-pathological adjacent liver tissues (Figure 2), indicating the valuable roles of these ncRNAs in tumorigenesis. 

Increased vtRNA1-1 levels were also connected to cell proliferation in other cancer cell lines. Burkitt lymphoma cells BL2 and BL41 in normal conditions either do not express vtRNA1-1 at all or at low levels. Ectopic over-expression, however, accelerated cell growth markedly and also enhanced Epstein–Barr virus (EBV) establishment [50]. The effect of vtRNA1-1 in proliferation was further investigated in breast cancer lines. The overexpression of vtRNA1-1 in MCF7 cells led to a doubling in cell proliferation rates [51]. In MCF7 cells, the nucleic acid binding protein named polypyrimidine tract-binding protein-associated splicing factor (PSF) interacts directly with vtRNA1-1 [51]. Along with its nuclear role in RNA splicing [52], PSF is also a transcriptional regulator that inhibits the transcription of the proto-oncogene G antigen 6 (GAGE6) [51]. The binding of vtRNA1-1 to the RNA-binding domain of PSF weakened the interaction on the DNA-binding domain, which results in GAGE6 repression release and the transcription of the proto-oncogene. Thus, PSF-vtRNA1-1 interaction increases GAGE6 expression, resulting in enhanced cell proliferation [51]. Altogether, these studies revealed that, in cancer cells, vtRNA1-1 is an important contributor to proliferation and its absence impairs cell growth, thus possessing the characteristics of an oncogene. 

### 2.2. vtRNA1-1 Is an Anti-Apoptotic Factor

**Figure 3:**
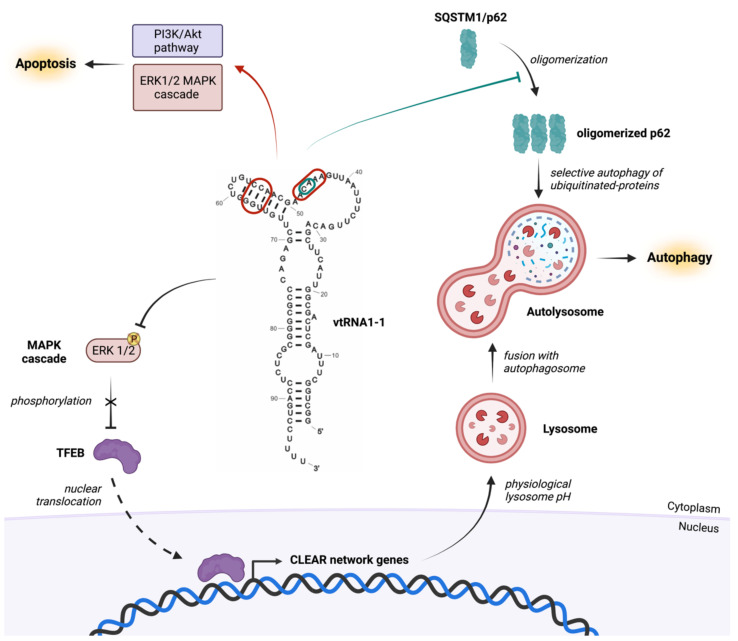
vtRNA1-1 regulates apoptosis and autophagy in cancer cells. In most studied cell lines (HeLa, BL41, BL2, A549, HEK293 and Hs578T), vtRNA1-1 is a pro-survival factor that inhibits apoptosis most likely through the regulation of the PI3K/Akt and ERK1/2 MAPK signaling pathways (red arrow). The important nucleotides in the central domain of vtRNA1-1 for apoptosis resistance are encircled in red [48,50]. In variance, in hepatocellular carcinoma cells (Huh-7), vtRNA1-1 does not affect apoptosis but positively regulates autophagy via the MAPK/TFEB signaling pathway that modulates the expression of the CLEAR network genes (black T-bars and arrows). This ensures the biogenesis of catabolically active lysosomes (acidic pH) capable of fusing with autophagosomes to form active autolysosomes [49]. Moreover, vaultRNA1-1 is able to inhibit p62 oligomerization and has been suggested to regulate the available cargo of ubiquitinated proteins for the autophagosome (green T-bar) [58,64]. The relevant residues on vtRNA1-1 for this proposed role are encircled in green.

Apoptosis is an essential physiological process for maintaining tissue cell homeostasis. Hence, it is not surprising that apoptosis and some pathologic conditions including cancer, in which the tissue grows unrestrained, are interdependent processes. Indeed, the apoptotic mechanism is often suppressed in tumorigenic processes [54,55]. Various cancer cell lines, having had the vtRNA1-1 gene silenced or knocked out, were more susceptible to apoptosis [48,50]. What is critically important is that the re-expression of the vault RNA1-1 rescued the resistance to apoptosis. Interestingly, hepatocellular carcinoma line Huh-7 is the sole cancer cell line investigated so far for which its apoptotic behavior is not regulated by vtRNA1-1 levels [49]. This mechanism of cell survival is stimulated upon vtRNA1-1 expression via the activation of PI3K/AKT (phosphoinositide-3-kinase–protein kinase B/Akt) and ERK1/2 MAPK (extracellular signal-regulated kinase 1/2 and mitogen-activated protein kinase) signaling pathways [48]. These two signaling cascades are well known pro-survival pathways that regulate many cellular mechanisms such as apoptosis as well as autophagy, protein synthesis, cell adhesion and migration [56,57]. The mechanism through which vtRNA1-1 confers resistance to apoptosis is still unclear, but a short stretch in the central domain of vtRNA1-1 has been identified as essential for the retention of its apoptosis resistance property (Figure 3) [48]. Mutational analysis revealed that the disruption of this short domain on vtRNA1-1, similarly to the vault RNA KO, increases apoptosis. This short stretch is located in the central region of the RNA, where the secondary structure features a single-stranded loop region and a stem-loop [48,58]. This domain has been shown to bind to p62 (sequestosome1 protein SQSTM1) [58] and is possibly also the anchor point for other vtRNA1-1 interacting proteins, although the binding partners of the vault RNA orchestrating apoptosis have not been uncovered yet. An initial mechanistic hint was recently published, suggesting a possible direct interaction of mouse vtRNA with MEK1, thus stimulating MAPK signaling and synaptogenesis [59]. If human vtRNA1-1 can fulfill a similar role in MAPK regulation as the sole murine vault RNA still needs to be shown. 

In Burkitt lymphoma cells, a link between vtRNA1-1 levels, EBV infection, NF-κB (nuclear factor kappa-light-chain-enhancer of activated B cells) activation and apoptosis resistance has been uncovered [50]. The EBV-encoded latent membrane protein (LMP1) stimulates the NF-κB pathway, resulting in the increased expression of vtRNA1-1, which led to an elevated expression of some known anti-apoptotic factors such as B-cell lymphoma extra-large protein (Bcl-xL) and the apoptotic protein with CARD (ARC). Thus, high vtRNA1-1 levels inhibit both the intrinsic and extrinsic apoptotic pathways [50]. It is noteworthy that this effect is vtRNA1-1 specific and independent from the vault particle since knocking down MVP had no effect on the apoptosis resistance phenotype in human B-cells. 

Cumulatively, the available evidence demonstrates that, with the exception of hepatocellular carcinoma [49], vtRNA1-1 plays a pivotal role in apoptosis resistance in several human cancer cell lines. 

### 2.3. vtRNA1-1 Regulates Autophagy and Lysosome Activity

Autophagy is a crucial mechanism for maintaining cellular homeostasis and is tightly interlinked to apoptosis [60]. Within cell homeostasis maintenance, autophagy has the catabolic role of degrading the unnecessary cellular material. Autophagy is operating in normal conditions for molecular clearance, but its activity is highly stimulated in situations of energy scarcity such as nutrient deprivation. The unnecessary or damaged cellular components that are not degraded by the proteasome are recycled through the autophagic process, which requires the fusion of autophagosomes to active lysosomes. Autophagy is particularly important in cancer. In the advanced stage of tumorigenesis, it can alleviate stress in the tumor’s microenvironment including hypoxic or nutritional stress [61]. On the other hand, autophagy acts as a tumor suppressor by clearing away damaged protein and organelles, which are potentially oncogenic molecules in the early stage of tumorigenesis [62].

p62 drives the identification of target cargos of the pre-autophagosome and initiates the autophagic process [63]. The oligomerization of p62 is necessary for its activity in the autophagic process. In liver cancer cells, evidence has been presented that vtRNA1-1 binds to p62 and interferes with its oligomerization, making vault RNA an inhibitor of p62-dependent autophagy (Figure 3) [64,65]. In nutrient rich conditions vtRNA1-1 suppresses autophagy, but under starvation conditions the steady-state levels of vtRNA1-1 decreases and autophagy becomes activated [64]. A more recent study provided a somewhat different and maybe more comprehensive perspective on the link between vtRNA1-1 and autophagy. In the hepatocellular carcinoma cell line Huh-7, the deletion of vtRNA1-1, or its reduction upon starvation, stimulated the ERK1/2-MAPK signaling pathway [49]. As a consequence, the phosphorylation of the transcription factor EB (TFEB), which is the master regulator of lysosomal biogenesis genes [49,66,67], becomes hyper-phosphorylated. Phosphorylated TFEB is unable to translocate to the nucleus; consequently, the CLEAR (Coordinated Lysosomal Expression and Regulation) gene network, which is under TFEB control, is suppressed and lysosome activity is downregulated. Of note, p62 is also under the transcriptional control of TFEB. Remarkably, lysosome pH measurements on vtRNA1-1 KO cells revealed a more alkaline lysosomal compartment resulting in severely impaired catabolic activities (Figure 3) [49]. Thus, it appears that the observed increased autophagic flux upon vtRNA1-1 deletion [64] is caused by impaired lysosomal autophagy-mediated clearance [49], explaining the accumulation of mature autophagosomes. In this mechanistic scenario, vtRNA1-1 is a positive regulator of autophagy.

### 2.4. vtRNA1-1 and Chemotherapy Drug Resistance

In tumors, the vtRNA1-1 paralogue usually has the lowest levels of promoter methylation and the highest chromatin accessibility [68]. Indeed, in cancer cell lines, vtRNA1-1 is commonly the most strongly expressed among the four vault RNAs [47]. What is particularly interesting is that the expression is increased in multidrug-resistant cells [46]. We have shown recently that Huh-7 cells lacking vtRNA1-1 expression had significantly impaired colony formation potential in vitro and possessed clearly reduced tumor growth capability in an in vivo xenograft mouse model [49]. Importantly, the treatment of Huh-7 cells or tumors of transplanted mice with Sorafenib, the first-line treatment option for hepatocellular carcinoma, was more potent in the absence of vtRNA1-1. This increased cytotoxicity of Sorafenib in the absence of vtRNA1-1 could be explained by the fact that less of the drug becomes trapped in the autophagosome, and the impaired lysosomes in the vtRNA1-1 KO cells cannot efficiently degrade the chemotherapeutic compound. Therefore, wild-type cells treated with equal concentrations of the drug are more resistant to its cytotoxicity compared to vtRNA1-1 KO cells [49]. Moreover, in several tumors such as glioblastoma, leukemia and osteosarcoma, vtRNA1-1 was proposed to be involved in Mitoxantrone resistance because it directly binds to the drug molecule hampering its efficacy [69,70].

Despite our still limited knowledge of vtRNA1-1 role in anti-tumor drug resistance, these findings might open new appealing therapeutic scenarios in the pharmacology field. The development of small molecules targeting vtRNA1-1 indeed could represent an important step in cancer treatment, being able to potentiate the anti-tumor effect of currently available drugs such as Sorafenib and Mitoxantrone.

## 3. vtRNA1-2, 1-3, 2-1 and Vault RNA-Derived Fragments

### 3.1. vtRNA1-2

The *VTRNA1-2* gene is flanked by *VTRNA1-1* and *1-3* genes [34,71] and is by far the least characterized vtRNA to date. This is not because it is assumed to have negligible cellular roles. On the contrary, its function is probably so crucial that it has been impossible so far to KO vtRNA1-2 to perform single-gene deletion analysis in human cell lines (our unpublished data). The most relevant information regarding vtRNA1-2 comes from The Cancer Genome Atlas data analysis, specifically the methylation state of its promoter [68,72,73]. The expression of vault RNA1-2 on average is suppressed in tumors compared to normal tissues according to the elevated level of promoter methylation [68,74,75]. This trend of gene silencing in neoplastic tissue is characteristic of tumor suppressor genes, and based on these data, it is reasonable to speculate about vtRNA1-2 being an anti-oncogene [68]. Nevertheless, in liver-derived metastasis, vtRNA1-2 levels are as high as the pro-proliferative vtRNA1-1 and, thus, significantly upregulated compared to the surrounding healthy liver tissue (Figure 2). This seemingly contradicting observation highlights the need for future dedicated research on this vtRNA paralogue. Moreover, vtRNA1-2 has a putative role in chemotherapy resistance since vtRNA1-2, like the 1-1 paralogue, binds directly to mitoxantrone, leading to its sequestration and decreased chemotherapy efficacy [69,70].

### 3.2. vtRNA1-3

The third vault RNA of the genomic locus 1 is an 88 nt long molecule. Unlike vtRNA1-1 and despite its apparent similarities in primary and secondary structures, the third paralogue does not regulate apoptosis or cell proliferation in Burkitt lymphoma or in HeLa cells [48,50]. In multidrug resistant cells, the expression level of vtRNA1-3 is increased and its association to the vault particle is also stimulated [46,47]. These observations suggest a possible role for vtRNA1-3 in drug resistance; however, the molecular mechanisms have yet to be determined. Furthermore, the hypermethylation of the vtRNA1-3 promoter in Myelodysplastic syndrome patients is correlated with decreased survival rates [76]. To date, vtRNA1-3 has been poorly studied; therefore, its potential role in cellular metabolism and cancer progression, for the most part, remains enigmatic.

### 3.3. vtRNA2-1

vtRNA2-1, previously known as pre-mir886 or nc886 [45], was the last small ncRNA to be identified as a vault particle-associated transcript [32]. vtRNA2-1, like the other three human vault RNAs, is encoded on chromosome 5 but in a separate genomic locus (Figure 1). The *VTRNA2-1* gene is transcribed into a ~100 nt long RNA by pol III even though the downstream B2-box motif is missing [32]. The fourth vtRNA regulates apoptosis and proliferation by interfering with the protein kinase R (PKR), which is a pro-apoptosis factor [45,77]. PKR is a double stranded RNA activated kinase; once activated, it regulates several signaling pathways such as NF-κB, PP2A, JNK and p38. Moreover, active PKR is able to phosphorylate the initiation factor eIF2α, thereby inhibiting further messenger RNA (mRNAs) translations [77]. vtRNA2-1 directly binds PKR, competing for its activation. Indeed, the proliferation in vtRNA2-1 knock-down (KD) cells is impaired due to unsuppressed PKR and eIF2α phosphorylation, which causes a general shut down of protein synthesis [45]. Unexpectedly, vtRNA2-1 levels were demonstrated to be suppressed (and hence PKR activity increased) in cholangiocarcinoma cells and in clinical samples from cholangiocarcinoma patients [78]. These findings suggest that vtRNA2-1 is involved in modulating PKR/eIF2α cell death pathways and tumorigenesis likely by ultimately activating the pro-survival NF-κB pathway. Moreover, very recently, vtRNA2-1 was reported for possessing a stimulatory effect on adenovirus gene expression and replication. It was proposed that vtRNA2-1 is involved in virus particle trafficking to the nucleus [79]. This observation is particularly interesting for the establishment of an anti-tumor therapy named “oncolytic virotherapy”, which is based on targeting proliferating tumor cells with cytolytic viruses, such as the adenovirus [79]. Furthermore, vtRNA2-1 expression was shown to be often epigenetically suppressed by DNA methylation in tumors, and this silencing correlated with poor outcome in patients affected by various cancers [80,81,82].

Based on these observation, vtRNA2-1 appears to have a tumor surveillance role. This could be employed as a prognostic marker in several cancers including lung, prostate, esophageal, gastric and acute myeloid leukemia [45,80,81,83,84,85].

### 3.4. vtRNA-Derived Fragments

The vast majority of vtRNA transcripts (~95%) do not associate with the vault complex and, thus, remain free or are associated with smaller RNPs in the cytoplasm (Figure 1). It was demonstrated that a small portion of this cytoplasmic vtRNAs (~2–5%) is subject to processing into smaller vtRNA-derived fragments. These processing events are Dicer-dependent, and the small vtRNA fragments likely represent miRNA-like molecules capable of mRNA silencing. Unlike canonical miRNAs, small vtRNA fragments are processed by Dicer from vtRNAs in a Drosha-independent manner [44,45,86,87]. A prerequisite for vtRNA Dicer cleavage is the introduction of a 5-methyl-cytosine (m^5^C), which is catalyzed by members of the RNA m^5^C methyltransferase NSUN family [44,88,89]. Via miCLIP (methylation individual-nucleotide-resolution crosslinking and immunoprecipitation), it has been determined that NSUN2 adds the modification on vtRNA1-1 at the position C69, on vtRNA1-2 at positions C27 and C59 and on vtRNA1-3 at C15, C27 and C59 [88]. Moreover, vault RNA1-2 is a specific target of NSUN1, which methylates the vtRNA at position C27 [89]. The m^5^C modification on vtRNAs was proposed to represent a regulatory molecular switch for Dicer processing [88]. Furthermore, the modification of m^5^C69 on vtRNA1-1 is prevented by the binding of serine/arginine rich splicing factor 2 (SRF2). The balance of NSUN2 and SRF2 binding to vtRNA1-1 orchestrates the regulation of its methylation and downstream vtRNA fragment production [90]. Once flagged with the m^5^C modification, the vtRNAs, similarly to pre-miRNA in miRNA biogenesis, are cleaved by Dicer. Small vtRNA-derived fragments associate with Argonaute proteins and guide gene expression regulation [10,44]. 

To date, the role of small fragments derived from the vtRNA1 locus is relatively unknown, but it has been shown that a vtRNA1-1-derived fragment is implicated in multidrug resistance in breast cancer. The ~23 nt vtRNA1-1 fragment targets and downregulates cytochrome CYP3A4 mRNA, an important drug-metabolizing enzyme [44,91,92]. Furthermore, another vtRNA1-1 fragment coordinates the epidermal differentiation of keratinocytes, although the exact molecular mechanism is still unclear [90]. 

More comprehensive insight has been uncovered on vtRNA2-1-derived fragments. At first, vtRNA2-1 was named pre-mir886 on account of its status of precursor for two small RNAs. Particularly studied is the vtRNA2-1-derived small RNA named miR-886-3p, which downregulates many key-features of neoplastic cells such as proliferation, migration, invasiveness in prostate, lung, and thyroid cancer [45,83,93]. Moreover, vtRNA2-1-derived fragments are less expressed in cancer compared to healthy tissue, and, overall, these observations are hinting towards a tumor-suppressor-like function [82,86,93,94]. On the contrary, in clear cell renal cell carcinoma (ccRCC), this very same vtRNA2-1 fragment was upregulated and stimulated the proliferation by inhibiting apoptosis [95]. In ccRCC, the miR-886-3p seed region targets the mRNA of the transcription factor PITX, a known tumor suppressor molecule, resulting in decreased PITX translation and reduced cell apoptosis [95]. However, this conflicting role of vtRNA2-1-derived fragments was not unanticipated since the vtRNA2-1 expression is inconsistent and regulated in a cancer type-specific manner. More precisely, it is upregulated or unaffected in the cancer of all tissues, except for kidney tumors [68]. Nevertheless, excluding renal cancer, overall miR886-3p, similarly to its precursor vtRNA2-1, suggests a tumor suppressor role. Therefore, more research has to be conducted in order to discriminate whether the anti-tumor effect of vtRNA2-1 is truly delivered by the full-length molecule or solely granted by its small RNA derivative. vtRNA2-1-derived processing products are the only ones so far that have also been linked to development. It has been suggested that a 24 nt long vtRNA2-1 fragment (called svtRNA2-1a) modulates early developmental processes in the central nervous system and cell type specification and is upregulated in brain areas affected by Parkinson’s disease [96].

Another intriguing role of small vtRNA fragments is the regulation of cell-to-cell communication, since they were abundantly found in exosomes [95]. Exosomes are small vesicles secreted by cells that can mediate cell–cell signaling, and along with other molecules can also transport RNA molecules [97]. There are many reports on exosome-based cell-to-cell communication carried out by miRNAs that participate in the regulation of tumorigenesis and angiogenesis [16,98,99,100,101]. It is, therefore, reasonable to speculate that those pathways can also be regulated by vtRNA fragments secreted via exosomes.

## 4. MVP and the Vault Complex: Implications to Cell Proliferation and Cancer

### 4.1. Cancer and Vault Complex

In this review, we set out to summarize the latest insights in vtRNA biology. Since vtRNAs are to a minor fraction also associated with the vault complex (Figure 1) and many publications did not disentangle the contribution(s) of unbound vtRNAs from vtRNAs associated with the vault complex, we also dedicate a chapter to the role of the major vault protein MVP and the entire vault RNP to cell proliferation and tumorigenesis. Thus far, only a few fragmentary functions of the vault complex have been identified including the assembly of the nuclear pore complex [102,103] or roles in innate immunity [104,105]. It was reported that the expression levels of the vault complex components such as vtRNAs and MVP varied in several cancer cells [46,68,83,85,86,106,107,108,109,110]. Furthermore, the intracellular localization of the vault complex tends to be changed from the cytoplasm to the nucleus in response to external stresses or stimuli, which are involved in tumorigenesis [26,111]. Because of this, the vault complex and its components are considered mediators of the nuclear-cytoplasmic translocation in both normal and cancer cells, albeit this appears to be controversial [26,112,113,114]. There are also a few studies connecting the vault complex to drug resistance [28,51,115,116,117,118,119,120,121,122], although it is still under dispute since reliable changes of drug resistance were not observed in in vivo experiments using MVP KO mice [26,123,124]. In addition to nuclear-cytoplasmic transport and drug resistance, it was reported that the vault complex participates in intracellular signaling pathways, DNA damage repair and anti-apoptotic processes, most of which are closely related to cancer [50,125,126,127,128,129].

### 4.2. Cancer and MVP

Among the three protein components of the vault complex [3,33], MVP is the most closely connected to tumor biology (Table 1). It has been reported that the MVP level is altered in various types of tumors. For instance, MVP is upregulated in lung tumor tissues compared to the adjacent normal lung tissues [109,130], as in the cisplatin-resistant lung adenocarcinoma cell line (A549/CDDP). The upregulation of MVP in lung cancer cells is related to interleukin 25 (IL-25) induction. Elevated IL-25 stimulates the expression of MVP and activates the several intracellular processes, including the NF-κB signaling pathway, contributing to chemotherapy resistances of lung cancer cells [130]. In opposition to these observations, MVP was also reported to exert tumor suppressor properties in Lewis lung carcinoma cells. The KD of MVP stimulated STAT3 (signal transducer and activator of transcription 3) and accelerated tumor growth. Moreover, the increased MVP expression in lung adenocarcinoma showed a better prognosis [109]. In prostate cancer, the upregulation of MVP expression was also considered as a putative prognostic biomarker of cancer. Contrary to lung cancer, in prostate cancer the elevated expression of MVP is associated with a more than 4-fold higher death risk [131]. In breast cancer cells, likewise, a high MVP level was associated with poor prognosis and the induction of chemotherapy resistant metastasis [132]. It is known that induced MVP expression by adipocytes could contribute to an MVP-related multidrug-resistance phenotype in breast cancer cells [133]. Moreover, the infections of hepatitis B virus and hepatitis C virus, which are known to increase the risk of hepatocellular carcinoma (HCC), elevate MVP expression. On the contrary, a deficiency of MVP inhibits HCC development induced by viral infection [134,135]. The overexpression of MVP is also observed in ovarian cancer and is linked to the development of multidrug resistance [136]. Thus, it can be concluded that MVP misregulation is clearly implicated in tumorigenesis, but the exact role of MVP during tumorigenesis varies depending on the cancer type. 

### 4.3. Drug Resistance and MVP

The architectural core of the vault complex structure is composed of 78 copies of the MVP, each possessing two Ca^2+^ binding sites that can interact with other proteins such as PTEN [137,138]. Through this binding, MVP and the vault complex can mediate diverse intracellular responses in various cell types including cancer cells. MVP in macrophages inhibits NF-κB signaling, thereby alleviating metabolic diseases [139]. The induction of MVP expression by viral infection upregulates type-I interferon production by enhancing the expression of IRF7 (interferon regulatory factor 7), but not IRF3 (interferon regulatory factor 3) [105]. Moreover, MVP inhibits the calcineurin-NFATc1 signaling pathway, which regulates genes involved in intracellular calcium concentrations, negatively regulating osteoclast differentiation and bone resorption [140]. MVP is also closely connected to signaling pathways in cancer cells, and upregulation most often results in chemotherapy resistance of the cells. In breast cancer, for instance, increased Notch1 upregulates MVP expression to activate the AKT pathway, which is involved in multidrug chemotherapy resistance and endothelial to mesenchymal transition promotion [132]. Another example is the relationship between MVP and B7-H3, a tumor-promoting glycoprotein [141]. The B7-H3 glycoprotein activates the MEK pathway through MVP-enhancing B-RAF, and the depletion of MVP inhibits B7-H3-induced MEK activation and stem cell propagation. The Ras pathway-independent regulation of B7-H3-induced stem cell propagation by the MVP-MEK signaling axis can be applied to develop novel strategies for overcoming cancer cell resistance to chemotherapy. Increased MVP expression caused by vtRNA2-1 induction by transcription factor E2F1 can also drive multidrug resistance in cervical cancer cells [24]. Additionally and particularly interesting for oncolytic virotherapy, vtRNA2-1 seems to play a central role in intracellular adenovirus trafficking [79]. Even though it is not investigated in this study, it is possible that the observed virus trafficking phenotype depends on the interaction of the adenovirus with the vault complex via vtRNA2-1 serving as an adaptor. Indeed, it has previously been observed that vault particles, similarly to the reported adenovirus particles [79], move along microtubules [142,143]. Moreover, in colon cancer cells, the interaction of MVP with miR-193a controls selective sorting into exosomes, regulating tumorigenesis [144]. This regulation of exosomes carried out by MVP indirectly affects the efficacy of chemotherapy treatment and the tumor progression of colon cancer. MVP has also been reported to contribute to temozolomide (TMZ)-resistance in glioblastoma [53]. The expression of MVP increased in glioblastoma with TMZ resistance, and its sensitivity to the drug was inhibited by increased MVP. In addition to the aforementioned effect, MVP is known to mediate multidrug resistance in a variety of cancer types including lung, ovarian and prostate cancers, although the effect is cell-type specific [28,119,122,123,131,136,145,146]. Therefore, the approach of targeting MVP for reducing multidrug resistance has the potential of being an effective and novel strategy for tumor therapy that could work synergistically with existing treatment regimes.

### 4.4. Proliferation, Apoptosis and MVP

Among all the signaling pathways that have been shown to have a connection to the MVP or the vault complex, proliferation and apoptosis are particularly important with respect to cancer. It was demonstrated that the MVP interacts with BAG3 (Bcl-2-associatged athanogene) to regulate potent pro-survival pathways including ERK signaling and contributes to chemotherapy resistance [147]. The depletion of either MVP or BAG3 inhibits the activation of ERK1/2, which in turn promotes adriamycin-induced apoptosis of breast cancer cells. MVP on the cell surface of hepatocellular carcinoma cells induced by several environmental stresses stimulates cancer progression [14]. The KD of MVP and treatment with anti-MVP antibodies reduced cell proliferation and induced apoptosis in HCC cells, indicating that cell-surface MVP negatively regulates cell proliferation and promotes apoptosis. In addition, MVP significantly enhances the aggressiveness of glioma cells based on MVP-mediated stabilization of the EGFR/PI3K signaling axis [148]. In glioblastoma, MVP expression was shown to be significantly increased and appears to be related to the malignancy of the tumor and to the survival rate of cancer patients [149]. In addition, MVP overexpression results in enhanced growth and brain invasion through the EGFR/PI3K signaling pathway in human glioblastoma xenograft models. Thus, MVP represents an interesting target for novel treatment approaches for brain cancer, including glioblastoma [150]. MVP is also related to the production of cytokines such as IL-6 or IL-8 upon viral infection or dsRNA stimulation and is associated with proliferation, apoptosis and chemotherapy resistance in several types of cancers, including colon cancer [151]. 

**Table 1:** MVP and cancer.

Cancer Type	Related Process	Potential Application	Reference
Prostate cancer	Multidrug resistance	Prognostic biomarker	Ramberg H et al. (2021) [131]
Ovarian cancer	Multidrug resistance	Biomarker of survivability in ovarian cancer patients	Zhao YN et al. (2016) [136]
	Multidrug resistance	Novel therapeutic strategy	Szaflarski W et al. (2013) [145]
	-	Biomarker for the combination therapy with 3’-C-ethynylcytidine(ECyd) and platinum.	Fukushima H (2014) [152]
Lung cancer	STAT3 signaling pathway	Novel therapeutic strategy and prognostic biomarker	Bai H et al. (2019) [109]
	NF-kB signaling pathway and IL-25	Clinical strategy overcoming the chemotherapy resistance	Shen W et al. (2019) [130]
	Doxorubicin resistance	Clinical strategies overcoming the doxorubicin resistance	Chen YL et al. (2016) [146]
	Apoptotic signaling mediated by immunosurveillance cytokines such as TRAIL	Novel therapeutic strategies for inflammation-mediated pathologies including cancer	Rayo J et al. (2021) [153]
	dsRNA or viral infection-induced expression of IL-6 and IL-8 by c-Fos and C/EBPβ	Regulating host pro-inflammatory response	Peng N et al. (2016) [151]
Breast cancer	Doxorubicin resistance	Novel therapeutic strategy for obesity-related chemoresistance	Lehuédé C et al. (2019) [133]
	BAG3 and ERK pathway	Novel therapeutic strategy	Pasillas MP et al. (2015) [147]
	B7-H3-induced stem cell propagation and MEK activation	Clinical strategy overcoming the chemotherapy resistance	Liu Z et al. (2019) [141]
	Notch1 signaling in TNBC	Clinical strategy overcoming the chemotherapy resistance	Xiao YS et al. (2019) [132]
Hepatocellular carcinoma	IRF2 and p53	Biomarkers of malignancy and survivability.	Yu H et al. (2020) [134]
	Cell-surface MVP (csMVP)	Malignancy biomarker and novel target for metastatic cancer	Lee HM et al. (2017) [108]
Glioblastoma	EGFR/PI3K signaling axis and PTEN	Novel therapeutic strategy for glioblastoma	Navarro L et al. (2015) [150]
	EGFR/PI3K signaling axis	-	Lötsch D et al. (2013) [148]
	Temozolomide resistance and survival rate	Novel therapeutic strategy for glioblastoma	Noh K.H et al. (2022) [149]
Colon cancer	Exosomal sorting of miR-193a	Novel therapeutic strategy	Teng Y et al. (2017) [143]
	-	Biomarker for the combination therapy with ECyd and platinum.	Fukushima H (2014) [152]
Cervical cancer	Transcription factor E2F1 and nc886 (vtRNA2-1)	Clinical strategies overcoming the chemotherapy resistance	Li JH et al. (2017) [115]
Osteoclasts	Calcineurin-NFATc1 pathway	Novel therapeutic strategy	Yuan L et al. (2021) [140]
Nasopharyngeal carcinoma	-	Biomarker for the combination therapy with ECyd and platinum.	Fukushima H (2014) [152]

## 5. Concluding Remarks and Open Questions

In conclusion, the four human vtRNAs as well as the vault complex and its major component, the MVP, are clearly related to various processes in cancer biology including proliferation, apoptosis and autophagy. Since their mechanistic contributions to tumorigenesis and chemotherapy-resistance remain poorly understood, further research is essential. One of the main open questions in the field that merits attention in the near future is the unclear interdependence of the vtRNAs and the vault complex. The fact that only a minor subpopulation of the vtRNA transcripts actually associate with the vault complex (Figure 1) supports the view that these ncRNAs possess biological roles that are independent of the vault complex. The most recent literature, as reviewed herein, seems to support the ribo-regulatory roles of these ncRNA molecules in cell proliferation, apoptosis resistance and tumorigenesis. Since mechanistic insights into vtRNA functions are still limited, identifying all the different protein interaction partners of vtRNAs in different cell types or in different phases of the cell cycle might solve yet another outstanding question: How can the four human vtRNA paralogs obviously orchestrate distinct (yet mainly pro-survival) signaling cascades in various cell types and tissues despite sharing significant sequence similarities? What determines specificity? Are different protein binding partners or distinct post-transcriptional vtRNA modifications at the heart of the functional specificities? How do vtRNA-derived fragments contribute to the observed phenotypes? Another central question that has not yet been addressed is about the driving force for maintaining vtRNAs and the vault complex in most eukaryal species (including mammals), while others (e.g., Drosophila, *C. elegans*) have apparently lost these genes during the course of evolution. Although many questions remain open and mechanistic insights into vtRNA biology are still limited, dedicated future research to uncover the hidden secrets of these interesting molecules is in our opinion justified. The apparent link between vtRNAs to cell proliferation, drug resistance and tumorigenesis make them promising candidates for novel therapeutic targets or diagnostic markers in several types of cancer.

## Data Availability

No new data were created or analyzed in this study. Data sharing is not applicable to this article.
